# The Hong Kong Early Child Development Scale: A Validation Study

**DOI:** 10.1007/s12187-012-9161-7

**Published:** 2012-10-03

**Authors:** Nirmala Rao, Jin Sun, Sharon Sui Ngan Ng, Kitty Ma, Yvonne Becher, Diana Lee, Carrie Lau, Li Zhang, Chun Bong Chow, Patrick Ip

**Affiliations:** 1 The University of Hong Kong, Pokfulam Road, Hong Kong, Hong Kong; 2 The Hong Kong Institute of Education, Tai Po, New Territories, Hong Kong, Hong Kong

**Keywords:** Early child development scale, Holistic development, Validation, Chinese

## Abstract

This paper reports on the development and validation of the Hong Kong Early Child Development Scale (HKECDS), a holistic measure of child development designed specifically for preschool children in Hong Kong. Scale development was an iterative process and the first version of the scale contained 190 items whereas the final version includes only 95. Children ranging in age from three to six years were administered trial versions of the HKECDS in Studies 1 (*n* = 60) and 2 (*n* = 240). Item analyses indicated that it is a developmental scale and that it has an appropriate level of difficulty for preschool children. It also discriminates between three- to six-year-olds from different social backgrounds in Hong Kong. The final version of the HKECDS includes items from the following eight subscales: Personal, Social and Self-Care (7 items), Language Development (13 items), Pre-academic Learning (27 items), Cognitive Development (10 items), Gross Motor (12 items), Fine Motor (9 items), Physical Fitness, Health and Safety (7 items), and Self and Society (10 items). The HKECDS is the first early child development scale which considers both the holistic development of preschool children and incorporates current expectations of early child development in Hong Kong. In this era of evidence-based decision making, it can be used to evaluate both the efficacy of targeted interventions and broader child-related public policies on early child development in Hong Kong.

The early years lay the foundation of life-long learning and development and three strands of research have led to increased attention to appropriate interventions in the prior-to-school years. First, research on early brain development indicates that the brain develops most rapidly in the first years of life and that environmental stimulation positively affects the developing brain (Shonkoff and Phillips 2000). Second, studies have documented a positive relationship between child care quality and child development outcomes (e.g., Cost, Quality and Child Outcomes Study Team 1995, NICHD ECCRN 2005; Sylva *et al.* 2006) and highlighted the importance of high quality early childhood services for children. Third, studies on the economic returns on investment in early childhood development have shown larger returns from government investment incurred in early childhood compared to adulthood (Heckman 2004; Lynch 2004).

A corollary to research that has documented the importance of the early years is that governments all over the world have been developing and enacting policies to enhance the well-being of young children, including improving preschool attendance and the quality of preschool education (Rao and Sun 2010, 2011). Hong Kong is no exception. Since preschool attendance is close to 100 % in Hong Kong (Hong Kong SAR Government 2011) and primary education is compulsory, there is less concern about a child’s readiness for school. Instead the government is committed to improving the quality of preschool education and ensuring that all children will have access to high-quality early education and care (Rao and Li 2009).

Researchers and policy makers in Hong Kong and elsewhere are keen to evaluate the sequel of both, specific interventions and broader social policies on early child development. Yet there is no tool which has adequate psychometric properties to conduct direct assessment of the holistic development of children. Against this background, we developed the Hong Kong Early Child Development Scale (HKECDS) and conducted two validation studies.

## Developing Contextually Appropriate Measures of Early Child Development

There are currently no globally accepted tests of early child development (ECD) primarily because of the concerns that assessments developed in one country (usually Western countries) may not be valid in other countries due to cultural and contextual differences in both, content and assessment techniques (Sun *et al.* 2012). However, contextually appropriate early child development tests are important for countries in their efforts to regulate and monitor early childhood services. Further, they can be used to analyze the impact of early childhood policies and programs on children, and track child development over time. Therefore, it is important to develop assessment tools that are relevant to a country’s specific goals for early child development and education.

In recent years, several countries in different parts of the world have exerted efforts to develop contextually appropriate measures of early child development. UNICEF in partnership with Columbia University launched the Early Learning and Developmental Standards (ELDS) project in 2002 to deal with the lack of appropriate instruments for assessing and monitoring children’s early development and learning. ELDS define what children are expected to know and expected to do. By 2009, culturally appropriate standards had been developed in 43 countries all over the world, including several Asian countries (Cambodia, China, DPR Korea, Fiji, Kiribati, Laos, Mongolia, the Philippines, Solomon Islands, Thailand, Vanuatu, and Vietnam). The use of standards operationalizes expectations for young children and provides a basis for measurement of young children’s learning (Kagan and Britto 2005). Results can be used to implement policy to promote more equitable learning outcomes.

All countries’ ELDS include aspects of physical, socio-emotional, cognitive and language development; no two nations’ ELDS are exactly the same since they reflect each country’s particular values and expectations of child development at specific ages. Based on their ELDS, several countries in the Asian region have developed instruments to assess child development, and validation studies have been conducted with nationally representative samples. These measures of child development include the Protocol to Assess Lao School Readiness Competencies (Rao 2008), Cambodian Developmental Assessment Test (Rao and Pearson 2007; Royal Government of Cambodia 2007), and Vietnam Early Learning and Development Checklist (Rao 2006). After the validation, these countries can use the results of child assessments in multiple ways including program and policy evaluation, tracking child development over time and to inform curriculum revision, instructional improvement, teacher training, parenting education and advocacy efforts (Miyahara and Meyers 2008). Hence it is clear that context-specific ECD assessment instruments are very useful.

A 192-item early child development scale was recently developed in Japan. Childcare professionals were invited to evaluate children’s competencies in the following domains: Gross motor, Fine motor, Social competence, Communication, Vocabulary, and Intelligence development. The sample included 22,819 children ranging in age from birth to 84 months enrolled in authorized child daycare centers across Japan and results indicated that the scale has satisfactory validity in measuring child development in Japan (Anme and Segal 2010).

The work on Hong Kong Early Child Development Scale (HKECDS) also represents an effort to develop and validate a territory-specific early child development scale. Our objective was to develop a measure that relied on direct assessment of children in lieu of teacher or parent reports and which reflected goals and expectations for ECD in Hong Kong.

## Holistic Early Child Development and Education in Hong Kong

Early childhood education is entering a very positive era in Hong Kong (Rao and Li 2009) as the government has given increased attention to early childhood education and made considerable progress in terms of improving its quality (Ng, Sun and Lau in press; Rao 2010). The values and expectations of early child development for Hong Kong’s young children are expressed explicitly in the Guide to the Pre-primary Curriculum (Curriculum Development Council, HKSAR 2006), “....to nurture children to attain all-round development in the domains of ethics, intellect, physique, social skills and aesthetics, so as to prepare them for life” as well as “to stimulate children’s interest in learning and cultivate in them positive learning attitudes, in order to lay the foundation for their future learning” (p.18). It should be noted that the Guide explicitly states that we should not expect 3-year-olds to write (p.95), and highlights that young children should not be over-burdened with academic pressure. As a result, it is clear that the Hong Kong government is now recommending a child-oriented and holistic development approach to early childhood teaching and learning, which is at odds with the traditional academic-oriented kindergarten curriculum which was typically followed in Hong Kong.

Following the developmental goals expressed in the Guide, six learning areas for children were identified (physical fitness and health, language, self and society, early mathematics, science and technology, and arts) to promote physical, cognitive and language, affective and social, as well as aesthetic development of young children in Hong Kong (Curriculum Development Council, HKSAR 2006, p.17). These specific values and expectations of early childhood development in Hong Kong guided the selection of domains of the HKECDS.

## Measurement of Holistic Early Childhood Development

Although considerable efforts have been exerted to improve the quality of early childhood education in Hong Kong, there is no psychometrically robust, culturally and contextually appropriate measure to directly assess the holistic development of young children in Hong Kong. The Wechsler Intelligence Scale for Children (WISC) has been validated with a Hong Kong sample, ranging in age from five to 15 years (Chen *et al.* 2010). However, the WISC focuses on children’s cognitive development and it is only suitable for children over 5 years. Further, test administration is limited to clinical and educational psychologists. Therefore, the WISC is not a good option to assess preschool children’s holistic development and/or monitor the quality of early childhood education in Hong Kong.

There are also measures developed to tap specific domains of early childhood development in Hong Kong. For example, The Hong Kong Cantonese Oral Language Assessment Scale (HKCOLAS) (Department of Health, HKSAR and Language Information Sciences Research Centre, City University of Hong Kong 2006) is the first assessment tool developed specifically for local speech therapists’ use in diagnosing language impairment in Hong Kong’s Cantonese-speaking pre-primary and primary school children. Again, such measurements which focus on only one domain are not appropriate tests of the holistic development of young children in Hong Kong.

Another important measure of early child development in Hong Kong is the Chinese Early Development Instrument (CEDI) (Ip 2012). The CEDI was developed based on the Early Development Instrument (Janus and Offord 2007), a population-based measure of child outcomes which has been used in many countries in the world. Teachers report on different dimensions of children’s school readiness and geographical mapping is used to identify vulnerable sectors within a city, region or country. The CEDI considers the school readiness of 5-year-olds in Hong Kong and relies on teachers’ reports of children’s performance. Both the CEDI and the HKECDS focus on children’s holistic development but the CEDI is only used for 5-year-olds and is a teacher-report measure. On the other hand, the HKECDS is suitable for children aged three to six and relies on direct assessment of child behavior rather than parent/teacher report. We chose to use a test as direct observations of child behavior have been shown to be more valid than parent/teacher reports (e.g., Lee *et al.* 2009).

There has been only one large scale empirical study of early childhood development in Hong Kong using a large and representative sample (Opper 1996) and the data were collected over 20 years ago. Hong Kong participated in the cross-national IEA (International Association for the Evaluation of Educational Achievement) pre-primary education study in 1987 (Montie *et al.* 2006) and a child development measurement appropriate for Chinese preschool children in Hong Kong was developed for this study. The Hong Kong component of the study included a representative sample of about 3,000 children, including 3-, 4- and 5-year-olds from 67 preschools in Hong Kong. The assessment instrument used by Opper (1996) included items in the following five domains: motor development, personal and social development, cognitive development, language development, and development of pre-academic skills (Opper 1996, p.11). While Opper reviewed tools to assess young children in Hong Kong, the majority of items were selected from instruments which had been developed and normed for children in Western countries. Only items which were deemed appropriate for preschoolers in Hong Kong were selected and modifications were made to make chosen items suitable for the Hong Kong context. For example, characters were used instead of letters in the language scale. Opper’s instrument served as the major source of items for the HKECDS (Trial Version 1).

## Research Objectives

Against this background, this study had the following objectives:

1. To develop a contextually appropriate measure of holistic development for children ranging in age from three to six years in Hong Kong.
2. To conduct validation studies to evaluate the psychometric attributes of the measurement scale.

It was assumed that the HKECDS would be a scale appropriate for the assessment of the holistic development of 3- to 6-year-olds in Hong Kong and that items would reflect developmental and educational expectations for young children. It was hypothesized that the HKECDS would be a developmental scale, with older children attaining higher scores in all domains than younger children. We also expected that socio-economic status (SES) would have an impact on children’s development in different domains due to the differences in learning opportunities and resources in both, family and preschool contexts for those from the high and low SES backgrounds based on the existing literature (e.g., Sirin 2005; Yeung *et al.* 2002). In addition, we expected that SES differences in the domains related to pre-academic skills would be more pronounced among younger children than the older ones since preschool attendance is almost universal for young children in Hong Kong, and preschool rather than school may act as “the great equalizer” in these domains.

## Study 1

In Study 1, we examined the appropriateness of the HKECDS (Trial Version 1) for children of different ages in Hong Kong.

### Methods

#### Participants

In Hong Kong, preschools are classified as non-profit making (about 85 % of preschools), or private independent kindergartens with the former charging lower fees and receiving subsidies from the government. All kindergartens in Hong Kong follow the Guide to the Pre-primary Curriculum mentioned earlier. We randomly selected one non-profit making kindergarten and one private independent kindergarten from lists of the names of these different types of kindergartens in Hong Kong in 2010. The principals from our first choice of kindergartens from each list agreed to participate in the study. Next, we randomly selected 20 children from each cohort of the two kindergartens, and obtained their parents’ consent for them to participate in this study. Our sample included 20 K1 children^1^ (11 girls), 20 K2 children (9 girls), and 20 K3 children (9 girls). Both kindergartens offered half-day programs as do the majority of kindergartens in Hong Kong. All participating children were Chinese in this study and they all understood Cantonese although some of them came from Mandarin-speaking families.

#### Measures

The HKECDS (Trial Version 1) was developed after a series of meetings among the authors who are researchers in the fields of early childhood education, special needs education, developmental psychology, and pediatrics. The meetings considered the appropriateness of the domains and of all items. Revisions to the content, procedures, or instructions for administering items were made after each meeting.

As noted earlier, the primary source of the items for the HKECDS (Trial Version 1) was the scale used by Opper (1992, 1996). At this stage, we included almost all direct assessment items used in Opper’s instrument (1996) (142 items) to examine the appropriateness of these items for our cohort of children. Children’s personal, social and self-care skills were examined *via* teacher or parent report in Opper (1996). We therefore transformed these items into direct assessment items (28 items). As items in Opper’s instruments (1996) were basically selected from tests used in the Western countries, we developed some new items with reference to the Guide to the Pre-primary Curriculum (Curriculum Development Council, HKSAR 2006) and relevant literature to better fit the Hong Kong situation and to reflect cohort effects and secular trends (20 items). Newly-developed items reflected new ways of conceptualizing school readiness (e.g., inclusion of items to assess approaches to learning) and new competencies referred to in the government’s 2006 pre-primary education curriculum. Because of these amendments to Opper’s measures, we assumed that the HKECDS (Trial Version 1) could be considered as a culturally and contextually appropriate assessment tool.

The items were first developed in English and translated into Cantonese by research assistants who were Chinese-English bilinguals. The accuracy of the translation was evaluated and changes were made, if necessary, by members of the research team who were very proficient in both languages. The administration manual was developed in English but detailed written information about administration procedures for sub-items was developed in Chinese.

The HKECDS (Trial Version 1) therefore included 190 items from the following eight subscales: Personal, Social and Self-care (24 items); Language Development (18 items); Pre-academic Learning (49 items); Cognitive Development (18 items); Gross Motor (28 items); Fine Motor (33 items); Physical Fitness, Health and Safety (10 items); and Self and Society (10 items). All 190 items were administered to each child, regardless of age.

#### Procedure

Two undergraduate and two graduate students majoring in early childhood education were trained to administer the HKECDS (Trial Version 1). Before formal data collection, the four assessors went through all the test items, instructions, and materials with the second author. They were also required to practice each item during the training session to ensure that all of them followed the standardized testing instructions and procedures. In addition, they engaged in pilot studies with children aged three to six to get familiar with the testing procedures and materials, and to double code children’s performance during assessment. They had to achieve an inter-rater reliability of about 90 % of agreement before starting formal data collection.

All assessments were conducted in a quiet room in children’s kindergartens in individual sessions. The assessor and the child tested were the only individuals in the room, which contained a child-sized table and two chairs for testing. Children were required to respond to the assessor’s questions or requests by providing verbal answers or with physical actions. For each item, the assessors used standardized stimuli and followed standardized instructions, procedures, and scoring rules. Gross motor activities were conducted either inside or outside the room, depending on the space available in the room. Children were given some time to get familiar with the environment and the assessor by playing with some toys or chatting with the assessor at the beginning of the session. They were told that they would be playing some games during the sessions.

The test was administered over two sessions, which lasted 30–45 min each, as all children were given all 190 items. The interval between the two sessions was 1 day as children attended half-day programs. If a child was absent the next day, he or she was administered the next part of the test as soon as he/she came back to school. All children received a sticker as a gift at the end of the second assessment session.

#### Approaches to Analyses

We conducted item analyses based on the Classical Test Theory (CTT) to look at the facility and discrimination of the scale (Kline 2005). Specifically, we examined (i) children’s responses to individual test items in the HKECDS (Trial 1 version) in order to assess the quality of those items and choose items of optimum difficulty; and (ii) the quality of the HKECDS as whole. The quality of individual items was assessed by examining children’s responses and determining whether or not each item was pitched at an appropriate level of difficulty and whether or not it differentiated among children with different levels of competencies. The quality of the test as a whole was evaluated by estimating its internal consistency.

The following steps were adopted in the analyses:

Step 1: The passing rates and mean scores for each item for children of different ages were calculated to derive an index of item difficulty. A higher value represented an easier question;
Step 2: Items that did not show extreme means, zero or nearly zero variances and represented a developmental trend with different ages of children were considered as items with optimum difficulty and were chosen (K3 > K2 > K1; if the passing rate for one age group was higher than 90 %, the passing rate for the older age group was not 100 %; if the passing rate for one age group was lower than 10 %, the passing rate for the younger age group was not 0 %; and at least the passing rate for one age group was higher than 10 % and lower than 90 %.);
Step 3: We calculated the internal consistency of each subscale using items which were retained after step 2 and chose internally consistent items which measure a unitary ability or attribute defined in the subscale (average inter-item correlation > .20; we expected an increase in Cronbach’s alphas after removing items that were not internally consistent with other items);
Step 4: We examined passing rates and mean scores for the remaining items. An index of difficulty was recalculated for each item. This resulting scale is referred to as the HKECDS (Trial Version 2).

However, there were some items which may not have met the inclusion criteria of passing rates and mean scores, but they were considered as tapping important abilities of Chinese children in Hong Kong. For example, knowledge of viral transmission is considered important for preschoolers in Hong Kong because of the SARS outbreak in Hong Kong in 2003 and the Swine flu pandemic which led to preschool and school closures. Hence, after thorough discussions among the research team, we decided to retain items tapping children’s knowledge on viral transmission in the scale, although children did not show a steady progression in terms of the passing rates in this item in Study 1.

The research team also evaluated the value of each sub-scale and appropriateness of each item in the scale for 3- to 6-year-olds in Hong Kong.

### Results

Analyses of the appropriateness of items in each subscale were conducted following the above-mentioned steps. Table 1 presents the number of items that did not meet the inclusion criteria. It also shows the number of items which remained in the scale after the passing rate/means score check but did not correlate well with other items in the subscale; the internal consistency of each subscale tended to increase if these items were deleted from it.

**Table 1:** Changes of number of items in each domain in the scale after item analyses in Study 1

Domains (A)	Initial number of items (B)	Number of items showing extreme variance (C)	Number of items not internally consistent with others (D)	Number of items retained in the scale despite not meeting the inclusion criteria (E)	Final number of items (B − C − D + E)
Personal, social and self-care	24	12	3	0	9
Language development	18	7	1	4	14
Pre-academic learning	49	20	2	2	29
Cognitive development	18	10	0	2	10
Gross motor	28	10	6	0	12
Fine motor	33	21	2	0	10
Fitness, health and safety	10	1	1	1	9
Self and society	10	1	0	1	10

As mentioned earlier, we included some items in the scale despite their low discrimination ability because of our belief that they reflected expectations for competencies which were important for Chinese preschoolers in Hong Kong. After examining the raw data and the instruction manual, we assumed that low discrimination indices for some items were due to ambiguous wording in instructions or problems in testing procedures. We therefore amended these items and clarified the wording in the instructions, improved the stimuli used, and/or improved the testing procedures. For example, we added a demonstration within the instructions for the item which required children to clip two sheets of paper together as we felt that children may not have passed the item because they were not familiar with how to use paper clips and not because they lacked the relevant fine motor abilities to complete the task. The number of such items in each subscale is indicated in Table 1.

We administered all items to children in K1, K2 and K3 in Study 1 to examine the age appropriateness of the items. This made the assessment very time-consuming and increased the cognitive burden especially for the younger children. Hence we felt the next version of the scale should adopt an alternative approach since we still wanted to use equivalent items for children of different ages. As a result, the items and sub-items in the HKECDS (Trial Version 2) were arranged according to difficulty level and discontinue rules were added in the scale when the items were administered to children of different ages. For example, in one pre-academic item on simple addition, four sub-items were included. All children started at the easiest sub-item but any remaining sub-items were skipped if the child was unable to give correct answers to two consecutive sub-items.

After the above steps, we ended up with the HKECDS (Trial Version 2) with 103 items (Cronbach’s alpha = .98) from eight domains of Personal, Social and Self-care (9 items, Cronbach’s alpha = .68); Language Development (14 items, Cronbach’s alpha = .84); Pre-academic Learning (29 items, Cronbach’s alpha = .91); Cognitive Development (10 items, Cronbach’s alpha = .82); Gross Motor (12 items, Cronbach’s alpha = .75); Fine Motor (10 items, Cronbach’s alpha = .79); Physical Fitness, Health and Safety (9 items, Cronbach’s alpha = .64); and Self and Society (10 items, Cronbach’s alpha = .70). The correlations among the sub-scales are shown in Table 2.

**Table 2:** Correlations among eight sub-scales of the HKECDS (Trial version 2)

	PSS	LD	PL	CD	GM	FM	PFHS	SS
PSS	1	.60^*^	.57^*^	.60^*^	.54^*^	.53^*^	.53^*^	.46^*^
LD		1	.76^*^	.77^*^	.47^*^	.66^*^	..73^*^	.71^*^
PL			1	.84^*^	.45^*^	.61^*^	.58^*^	.71^*^
CD				1	.46^*^	.64^*^	.67^*^	.63^*^
GM					1	.50^*^	.45^*^	.33^*^
FM						1	.59^*^	.42^*^
PFHS							1	.60^*^
SS								1

*PSS* Personal, Social and Self-care; *LD* Language Development; *PL* Pre-academic Learning; *CD* Cognitive Development; *GM* Gross Motor; *FM* Fine Motor; *PFHS* Physical Fitness, Health and Safety; *SS* Self and Society. ^*^ *P* < .05

A MANOVA (Multivariate Analysis of Variance) with gender (2) × age (3) × kindergarten (2) as independent variables and subscale scores as dependent variables indicated that the main effect of kindergarten type was significant for some subscales, hence kindergarten was treated as a covariate in subsequent analyses. The MANCOVA, looking at children’s performance in different subscales with age (3) and gender (2) as the independent variables and kindergarten (2) as the covariate, yielded significant main effects of age in the omnibus analyses, *F* (16, 82) = 7.23, *p* < .001, *η*_*p*_^*2*^ = .59. Since we did not find significant gender differences in the analyses, the data for boys and girls were collapsed in the following analyses. The follow-up univariate analyses showed significant main effects of age in all subscales, with older children showing better performance than the younger ones (see Table 3). These results indicate that all the subscales of the HKECDS (Trial Version 2) are developmental in nature as children obtain higher scores on each subscale as they mature.

**Table 3:** Performance of children of different ages on the HKECDS (Trial version 2) in Study 1

Domains	K1 classes *M (SD*)	K2 classes *M (SD*)	K 3 classes *M (SD*)	F(2, 48)	*η*_*p*_^*2*^
Personal, social and self-care	13.03 (.96)^a^	14.91 (.91)^a^	19.00 (.91)^b^	10.80^*^	.31
Language development	23.33 (.84)^a^	29.44 (.80)^b^	33.18 (.80)^c^	36.88^*^	.61
Pre-academic learning	22.99 (1.99)^a^	38.12 (1.90)^b^	53.99 (1.90)^c^	67.62^*^	.74
Cognitive development	18.77 (.78)^a^	23.01 (.75)^b^	27.20 (.75)^c^	30.34^*^	.56
Gross motor	15.20 (1.09)^a^	18.66 (1.04)^ab^	21.33 (1.04)^b^	8.29^*^	.26
Fine motor	14.41 (.95)^a^	19.11 (.91)^b^	24.01 (.91)^c^	26.54^*^	.53
Physical fitness, health and safety	4.25 (.40)^a^	6.59 (.38)^b^	7.40 (.38)^b^	17.15^*^	.42
Self and society	6.48 (.59)^a^	10.47 (.56)^b^	12.07 (.56)^b^	25.05^*^	.51

Means that in the same row that do not share subscripts differ in the Bonferroni Test. ^*^ *P* < .05

In summary, the HKECDS (Trial Version 2) showed acceptable difficulty and discrimination to measure the intended child development outcomes in different domains. The sub-scales of the HKECDS (Trial Version 2) showed medium level of inter-correlations. This indicated that while each sub-scale measured different abilities of child development, the full scale provided a measure of holistic child development. Further, the HKECDS (Trial Version 2) was more concise than the original version and the contents tapped were considered to reflect the expectations of child development in current Hong Kong society. Discontinue rules were added to the HKECDS (Trial Version 2) to maintain item equivalence while reducing the cognitive burden for children with lower level of abilities at the same time. However, as a society with a dramatic gap between the rich and the poor (Pang and Lau 2007), we wished to determine whether or not the HKECDS (Trial Version 2) would be appropriate for children from different family backgrounds. Hence, in Study 2, we particularly examined the reliability and validity of the HKECDS (Trial Version 2) when it was administered to children who were from both low- and middle-class families.

## Study 2

In Study 2, we administered the HKECDS (Trial Version 2) to children from different family backgrounds. However, all children were from kindergartens in which preschool education vouchers could be encashed and this is true of 85 % of kindergartens in Hong Kong. These kindergartens have fees below HKD 24,000 per year for half-day programs or HKD 48,000 per year for a full-day session.

### Methods

#### Sampling Approaches

Given variations in income levels across different districts in Hong Kong, we decided to first locate areas where we would recruit the participants with contrasting family backgrounds. Two areas, Central (Central and Western District) and Tin Shui Wai (Yuen Long District), were selected accordingly. Central and Western District is one of the most expensive districts in Hong Kong and family income is higher in this district than in others. Hence, we assumed that children enrolled in kindergartens in Central would be from middle-class families. On the other hand, the majority of residents in Tin Shui Wai are immigrants from mainland China or blue collar workers whose family income is quite low compared to families in other districts.

We drew up lists of kindergartens in the two areas of Central and Tin Shui Wai and randomly selected one kindergarten in each area first, and then sought the principal’s consent. After the numbers of children in each age group who could participate in this study were confirmed, we again randomly selected another kindergarten until we obtained consent from parents of the desired number of children. Four kindergartens were therefore selected to participate in this study after such a process. It should be noted that all the kindergartens we approached agreed to participate in the study. The kindergarten in Central provides half-day programs and the school fees were around HKD 23,600 per year. The kindergartens in Tin Shui Wai provide both half-day and full-day programs and the school fees were about HKD 14,000 per year for the half-day programs and HKD 26,000 per year for the full-day programs. Hence the fees for the Central kindergarten were almost double that of those in Tin Shui Wai.

#### Participants

A total of 240 Chinese children (49 K1, 49 K2, and 142 K3 children) from four kindergartens with one in Central district and three in Tin Shui Wai participated in Study 2 which took place in 2011.

Since the data collection in Study 2 was combined with another project on school readiness among Hong Kong preschool children 1 year before they entered primary school, teachers of the K3 children in Study 2 were also surveyed on children’s school readiness and we had to recruit a larger sample of K3 children than children in other age groups to meet the sample size requirement for the survey study. Information about the participants is provided in Table 4.

**Table 4:** Participant characteristics in Study 2

Kindergartens	Area	K1 classes (3- to 4-year-olds) (# of boys)	K2 classes (4- to 5-year-olds) (# of boys)	K 3 classes (5- to 6-year-olds) (# of boys)	Total (# of boys)
Kindergarten 1	Central	24 (2)	24 (4)	70 (9)	118 (15)
Kindergarten 2	Tin Shui Wai	25 (13)	25 (17)	38 (23)	88 (53)
Kindergarten 3	Tin Shui Wai	–	–	16 (7)	16 (7)
Kindergarten 4	Tin Shui Wai	–	–	18 (9)	18 (9)

#### Measures

The HKECDS (Trial Version 2) validated after Study 1 was adopted in Study 2. As mentioned earlier, the HKECDS (Trial Version 2) included 103 items from eight domains of Personal, Social and Self-care (9 items); Language Development (14 items); Pre-academic Learning (29 items); Cognitive Development (10 items); Gross Motor (12 items); Fine Motor (10 items); Physical Fitness, Health and Safety (9 items); and Self and Society (10 items).

#### Procedures

Three research assistants administered the HKECDS (Trial Version 2) in Study 2. Two of them had been involved in the data collection for Study 1. All assessors again went through all the testing items and procedures with the second author. They practiced the HKECDS (Trial Version 2) in one to two sessions with children of different ages before conducting the actual data collection. An inter-rater reliability of 90 % was achieved among the assessors in the practice sessions.

All children completed the test in one session which took about 45 min. The eight subscales of the test were administered in a quiet classroom in the kindergarten except for the gross motor subscale which was given outside the classroom due to limited space. Children received a sticker as a gift when they finished the test.

#### Approaches to Analyses

As in the case of Study 1, we conducted item analyses by examining children’s passing rate/mean score for each individual item to determine the age appropriateness and difficulty level of the items and examined the internal consistency of the subscales and the scale as a whole. We also conducted several meetings among members of our research group to further look at the structure and items of the revised scale and to make necessary revisions for problematic items.

We then compared the performances of children of different ages, and from different family backgrounds for each subscale to examine the sensitivity of the further revised HKECDS (Trial Version 3), to evaluate possible ability differences of children. We refer to HKECDS (Trial Version 3) as the HKECDS.

### Results

We examined passing rates/mean scores for each item and found that nine items showed extreme variance (extreme means near 0 or 100 % of the passing rates). These items did not differentiate among children and eight of them were removed from the scale (Personal, Social and Self-care: 2 items; Language Development: 1 item; Pre-academic Learning: 2 items; Fine Motor: 1 items; and Physical Fitness, Health and Safety: 2 items). One item (using chopsticks) which did not differentiate among children with different abilities was retained for the subsequent version of the HKECDS. This is because we consider the skills of appropriately using chopsticks as an important and unique component of Personal, Social and Self-care abilities for Hong Kong-Chinese children. However, administrative procedures for this item were amended in the next version of the scale in an attempt to enhance its discriminative ability.

The internal consistency of each subscale was also examined after the above mentioned items/sub-items were removed from the scale. All remaining items had high correlations with other items in the subscale, and the Cronbach’s alphas of the subscales would all be reduced if the remaining items were deleted. These indicated the satisfactory internal consistency of the subscales and that the individual items were measuring similar ability to other items in the same subscale.

As a result, the latest version of the HKECDS has a total of 95 items (*α* = .97) with eight subscales. In the 95-item scale, 55 items are derived from Opper (1996) but we amended the scoring criteria in a few cases so all sub-items are scored on a 0/1 basis. The rest of the items were developed by us. Further, detailed instructions, procedures, and scoring criteria were provided for all newly-developed items. Detailed information about each subscale and examples of items are presented in Table 5. Table 6 presents the inter-correlations among the scales.

**Table 5:** Examples of items in the HKECDS

Domains	# of items	Cronbach’s alphas	Sample items
Personal, social and self-care	7	.63	Puts on the smock by him/herself
			Tells his/her birthday
Language development	13	.80	Using four pictures, tells a story which represent a logical sequence
			Identifies use of different objects
Pre-academic learning	27	.95	Counts in 10s from 40 to 100
			Measures a pencil
Cognitive development	10	.70	Predicts patterns
			Understands symbols and concepts
Gross motor	12	.78	Kicks a stationary ball
			Walks forward heel-to-toe along straight line
Fine motor	9	.75	Ties a single knot
			Makes a bowl out of playdoh
Physical fitness, health and safety	7	.61	Understands daily servings in food pyramid
			Understands methods to prevent germ transmission
Self and society	10	.64	Distinguishes between recyclable and non-recyclable materials
			Recognizes national and regional flags

**Table 6:** Correlations among eight sub-scales of the HKECDS (Trial version 3)

	PSS	LD	PL	CD	GM	FM	PFHS	SS
PSS	1	.67^*^	.72^*^	.65^*^	.39^*^	.42^*^	.50^*^	.62^*^
LD		1	.77^*^	.73^*^	.40^*^	.48^*^	.69^*^	.67^*^
PL			1	.79^*^	.43^*^	.56^*^	.63^*^	.73^*^
CD				1	.37^*^	.47^*^	.62^*^	.69^*^
GM					1	.48^*^	.29^*^	.44^*^
FM						1	.38^*^	.43^*^
PFHS							1	.57^*^
SS								1

*PSS* Personal, Social and Self-care; *LD* Language Development; *PL* Pre-academic Learning; *CD* Cognitive Development; *GM* Gross Motor; *FM* Fine Motor; *PFHS* Physical Fitness, Health and Safety; *SS* Self and Society. ^*^ *P* < .05

**Fig. 1:**
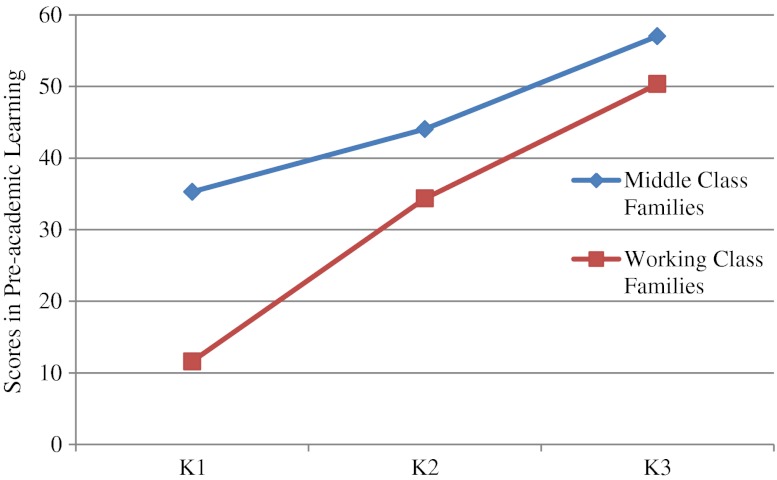
Performance of children from different family backgrounds on the HKECDS in the domain of Pre-academic Learning in Study 2

**Fig. 2:**
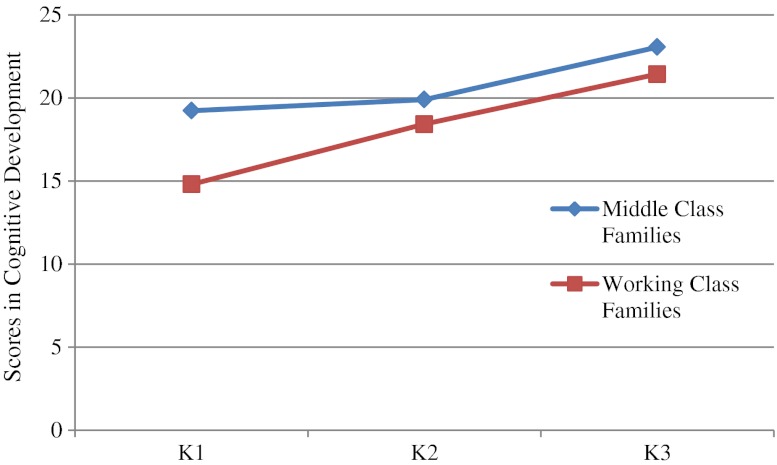
Performance of children from different family backgrounds on the HKECDS in the domain of Cognitive Development in Study 2

We further examined the ability of the HKECDS to discriminate among children of different ages and from different family backgrounds. A MANOVA with age (3), family background (2) and gender (2) as between-subject variables was conducted to examine children’s performance in each subscale of the HKECDS (Trial Version 3). There were significant main effects of age, *F* (16, 442) = 24.40, *p* < .001, *η*_*p*_^*2*^ = .47, and family background, *F* (8, 221) = 17.40, *p* < .001, *η*_*p*_^*2*^ = .39, and the Family Background × Age interaction was significant in the omnibus analyses, *F* (16, 442) = 3.14, *p* < .001, *η*_*p*_^*2*^ = .10. However, this time the main effect of gender was significant, *F* (8, 221) = 4.60, *p* < .001, *η*_*p*_^*2*^ = .14. Since the significant gender effects were only found in the domains of Pre-academic Learning, *F* (1, 228) = 11.20, *p* < .01, *η*_*p*_^*2*^ = .05, and Fine Motor, *F* (1, 228) = 6.24, *p* < .05, *η*_*p*_^*2*^ = .03, and the effect sizes for gender were very small, the data for boys and girls were pooled together again for subsequent analyses. The follow-up univariate analyses confirmed significant main effects of age and family background on all subscales. Older children performed better than the younger ones and middle class children performed better than those from working class families in all domains except Gross Motor and Fine Motor (see Tables 7 and 8). The follow-up univariate analyses also yielded significant Family Background × Age interactions in the domains of Pre-academic Learning, *F* (2, 228) = 15.53, *p* < .001, *η*_*p*_^*2*^ = .12, and Cognitive Development, *F* (2, 228) = 3.69, *p* < .05, *η*_*p*_^*2*^ = .03. As shown in Figs. 1 and 2, as children mature, the differences in performance between children from low-income and middle-class families in these two domains decrease.

**Table 7:** Performance of children of different ages on the HKECDS in Study 2

Domains	K1 classes *M (SD*)	K2 classes *M (SD*)	K 3 classes *M (SD*)	F (2, 228)	*η*_*p*_^*2*^
Personal, Social and Self-care	10.23 (.45)^a^	12.29 (.38)^b^	14.45 (.23)^c^	38.83^*^	.25
Language Development	20.17 (.93)^a^	25.30 (.77)^b^	29.31 (.48)^c^	40.92^*^	.26
Pre-academic Learning	23.44 (1.36)^a^	39.19 (1.13)^b^	53.67 (.69)^c^	217.14^*^	.66
Cognitive Development	17.01 (.48)^a^	19.16 (.40)^b^	22.25 (.25)^c^	56.60^*^	.33
Gross Motor	11.54 (.96)^a^	16.52 (.79)^b^	17.65 (.49)^b^	16.17^*^	.12
Fine Motor	10.31 (.76)^a^	13.37 (.63)^b^	18.05 (.39)^c^	50.16^*^	.31
Physical Fitness, Health and Safety	7.12 (.50)^a^	9.32 (.42)^b^	10.33 (.26)^b^	16.55^*^	.13
Self and Society	6.87 (.61)^a^	11.97 (.51)^b^	14.02 (.31)^c^	54.29^*^	.32

Means that in the same row that do not share subscripts differ in the Bonferroni Test. ^*^ *P* < .05

**Table 8:** Performance of children from different family backgrounds on the HKECDS in Study 2

Domains	Working class families *M (SD*)	Middle class families *M (SD*)	F (1, 228)	*η*_*p*_^*2*^
Personal, social and self-care	11.76 (.23)	12.89 (.36)	7.21^*^	.03
Language development	22.98 (.47)	26.88 (.73)	20.18^*^	.08
Pre-academic learning	32.10 (.68)	45.44 (1.06)	111.48^*^	.33
Cognitive development	18.22 (.24)	20.73 (.38)	31.45^*^	.12
Gross motor	16.36 (.48)	14.12 (.75)	6.36^*^	.03
Fine motor	14.63 (.38)	13.19 (.60)	4.10^*^	.02
Physical fitness, health and safety	8.05 (.25)	9.79 (.39)	13.84^*^	.06
Self and society	9.77 (.31)	12.13 (.48)	17.07^*^	.07

^*^ *P* < .05

## General Discussion

This paper describes the process of development and validation of the Hong Kong Early Child Development Scale (HKECDS). Results indicate that the HKECDS is a psychometrically robust, culturally and contextually appropriate measure of holistic child development for children ranging in age from three to six years in Hong Kong. A large number of items in the HKECDS were adopted and revised from the measure used in Opper (1996), the only large scale study on early childhood development in Hong Kong and data were collected over 20 years ago. Newly developed items were also added to reflect the cohort changes and current expectations for early child development with reference of the Guide to the Pre-primary Curriculum (Curriculum Development Council, HKSAR 2006). Although the instrument structure is similar with those in the Western countries, items in the HKECDS also tap culturally-sensitive expectations in each domain. For example, the coordination of fingers is considered important for fine motor development. The HKECDS examines young children’s development of such skills in a chopstick task, which is a common but unique activity in the Chinese culture. Other examples are items requiring children to match food with the Chinese festivals, name local landmarks and name the users of the four different colored Octopus cards (Child, Adult, Elder, Personalized). It is for these reasons that we argue that that HKECDS is a cultural and contextual appropriate measure of early child development in Hong Kong.

Two studies were conducted to examine the reliability and validity of the HKECDS with samples of children from different types of preschools and family backgrounds. The final version of the scale contains 95 items from the eight domains of Personal, Social and Self-care; Language Development; Pre-academic Learning; Cognitive Development; Gross Motor; Fine Motor; Physical Fitness, Health and Safety; and Self and Society. There was high internal consistency for each subscale of the HKECDS (range from .61 to .95). There were moderate inter-correlations among sub-scales which assess theoretically different constructs. Content validity was achieved *via* intensive discussions among the research group members from different fields related to child development and *via* careful revisions based on the discussions and the results from two waves of empirical studies. We therefore believe that the HKECDS assesses children’s holistic development in the Hong Kong context.

As predicted, older children attained higher scores than younger children in all subscales. This indicates that the HKECDS is a developmental scale that captures differences in children’s competencies as they mature. However, we did not find significant gender differences on the HKECDS. This finding is not in keeping with nation-wide, large-scale studies of school readiness in Canada and Australia which used the Early Child Development Index, a teacher-completed rating scale of child development at school entry which includes five developmental domains (Physical Health and Wellbeing, Social Competence, Emotional Maturity, Language and Cognitive Development and Communication Skills and General Knowledge). Results from the studies conducted in Canada (Janus and Offord 2007) and in Australia (Centre for Community Child Health and Telethon Institute for Child Health Research 2009) show that girls had higher scores than boys in all five domains. Our findings are somewhat consistent with Opper (1996) who found no significant gender differences in the areas of gross and fine motor development. She reported main effects of gender favouring girls for the overall sample in the areas of cognitive development, language development, and pre-academic development but follow-up tests revealed no significant differences at any of the three age levels. However, there were significant gender differences favouring girls in some aspects of social development (social interaction and social competency at all three ages and self-care at age 3) (Opper 1996). Further studies are needed to better understand the reasons for our findings but they may be related to more support being provided to boys.

Children from middle class families showed higher performance than those from working families in all subscales except Gross Motor and Fine Motor. In these two subscales, working class children performed better than those from the middle class families. These results are consistent with studies which have examined the influences of family background on early child development (e.g., Sirin 2005) and achievement gaps between children from high and low SES families in developed and developing countries (e.g., Moore *et al.* 2008, NICHD ECCRN 2000). Such differences in children’s performance may reflect the differences in learning opportunities and resources in both, the home and preschool contexts (e.g., Yeung *et al.* 2002). Children from higher SES background typically attend kindergartens which charge higher school fees and which have better qualified teachers (a proxy for preschool quality) than children from working class backgrounds. They also have more resources to facilitate their learning at home than children from working class families. Therefore, children from middle class families are involved in more learning-related activities and therefore show higher competencies in the domains of cognitive development, socio-emotional, pre-academic, and self and society than children from less socially advantaged backgrounds. In contrast, children from working class families may have more opportunities to play outside and help their parents with housework compared to other children and these experiences may have benefited the development of their gross and fine motor abilities.

As predicted, we also found that the SES differences in pre-academic learning and cognitive development were less pronounced in the K3 (5 year-old) children than in the K1 (3-year-old) children. As mentioned earlier, preschool attendance is almost universal for young children in Hong Kong and there is a tradition of academic-orientation in Hong Kong’s kindergartens despite the fact that government has exerted much effort to promote child-centered, less didactic early childhood education. In most countries, there are achievement gaps between children from low SES families and their more socially advantaged peers. Further, Grade 1 of primary school is typically considered “the great equalizer” as children from different family backgrounds experience the same curriculum. However, in the Hong Kong context, wherein preschool attendance is universal and the preschool curriculum is rather academic, this role is performed by preschools and we see a decrease in achievement gaps between K1 and K3.

### HKECDS and School Readiness

The concept of school readiness concerns children’s preparedness for school and typically includes cognitive and language development, physical well-being, psychomotor skills, socio­emotional development and approaches to learning. More comprehensive definitions of school readiness consider it to be a holistic concept which includes not only children’s readiness for school but schools’ readiness for children, families’ readiness to assist children to transition to primary school and interventions such as Head Start. Our study was concerned with children’s development and their developmental readiness. While we did not focus on school readiness *per se*, it is a component of developmental readiness. In many countries, children from economically disadvantaged backgrounds show poorer school readiness skills, which are considered critical for their later school success, than other children (Rouse *et al.* 2005). Children from low income families have fewer educational resources available at home than those from wealthier families, and schooling in many countries does not appear to close the gap. It is noteworthy that preschool education seems to be narrowing this “achievement gap” in the Hong Kong context.

The 95-item scale has a satisfactory level of difficulty, high internal consistency, good abilities to discriminate among children of different ages and varying family backgrounds, and sub-scales which adequately assess major domains of holistic child development. Although the majority of the items in the HKECDS were derived from Opper (1996), these were validated after two empirical studies and necessary revisions in terms of assessment procedures and instructions were made. Around half of the items were newly developed to reflect cohort changes and the current expectations for child development in the Hong Kong context. In addition, we assessed children’s development using tasks which are sensitive to Hong Kong’s Chinese cultural context and the experiences of preschool children. As a result, we believe that the HKECDS is a culturally and contextually appropriate early child development measure for Hong Kong.

With more and more attention and resources being allocated to early childhood education, the assessment of early child development in the region has become an urgent but difficult task for practitioners, researchers, and policy makers. The HKECDS is therefore expected to contribute to understanding and improving the quality of ECD research in Hong Kong because of its robust psychometric characteristics, and its conceptions of early child development in Hong Kong. In addition to domains such as cognitive development, socio-emotional development, and motor development, the HKECDS considers children’s knowledge and capacities in terms of self-care, physical fitness and health, and the local society. Such knowledge and skills are important for children today. Although there might be different expectations for child development among families with different backgrounds, these additional domains are consistent with the child developmental goals expressed in the Guide to the Pre-primary Curriculum and therefore could be considered as reflecting the expectations for young children in Hong Kong.

It should be noted that the HKECDS is more a tool to understand developmental levels in different domains of a group of children as a whole, rather than to determine the level of developmental functioning of an individual child. No decisions about individual children should be made based on the results of the HKECDS. Therefore, there is more tolerance of measurement errors and less stringent requirement for assessor training in scales such as the HKECDS than there is individual administered intelligence tests (Fernald *et al.* 2009). Early childhood educators or those with training in early childhood education can become qualified to use the HKECDS after they undergo specialized training. This enhances the possibilities of using the HKECDS as a comprehensive tool to understand, monitor, and track early child development in Hong Kong.

There are several limitations in this study. First, the item analyses we conducted were based on the Classical Test Theory (CTT), which has several limitations in comparison to the Item Response Theory (IRT) due to its inter-dependence of item and participants. Specifically, unlike IRT, the CTT approaches have inconsistencies of estimates of item difficulty in various samples, instability of difficulty indices across forms of tests, changes in internal consistencies across samples, and more measurement errors than the IRT approach (Magno 2009).

The second limitation of this study arises from its sampling procedures and sample size. We did not get a representative sample of preschool children in Hong Kong. However, we recruited children from different types of kindergartens and from different family backgrounds in the pilot studies to increase the diversity of participants. The sample sizes in the two pilot studies were quite small. In Study 2, we actually used the economic status of a neighborhood to index the family background of children recruited in this area. Although this makes sense intuitively, there was a lack of rigor in the process which may have led to bias in the results. Furthermore, the limitations in sampling strategies and sample size also prevented us from adopting confirmatory factor analyses to examine the construct of the scale and from employing IRT models for more comprehensive item analyses. Therefore, future studies with the scale should use a larger and representative sample of young children in Hong Kong to further examine the psychometric properties of the HKECDS.

Another limitation of the scale is the age range of children studied. The HKECDS focuses on children aged from three to six but neglects the under threes. The period from birth to three years is an important period in human development but there are significant differences in the developmental tasks for the under threes and for young children aged three to six. It is difficult to include the under threes in the current construct of HKECDS. But further work should examine the holistic development of the under threes.

Despite the limitations, the HKECDS is the first scale developed to directly examine the holistic early development and learning of young children in Hong Kong and considers the current expectations of ECD in the Hong Kong society. It taps multiple domains of early child development and learning and has an equivalent measurement structure for multiple age groups of children. The HKECDS relies on direct behavioral assessment of children. Although more resource intensive, such measures yield more reliable information about child development than those that typically rely on adult report (e.g., Lee *et al.* 2009).

There has only been one empirical study examining early child development in Hong Kong using a large and representative sample and this was conducted in 1987–1988 (Opper 1996). There are clear differences between children who attended preschool 25 years ago and those who attend preschool today. Research on child development should ideally be the basis of educational policy and practices affecting children and the HKECDS can be used to provide policy relevant information about early child development for researchers, policy makers, and educators in Hong Kong and perhaps further afar.

## Footnotes

1 There are usually three classes in kindergartens: K1 is for 3- to 4-year-olds; K2 is for 4- to 5-year-olds; K3 is for 5- to 6-year-olds.

## References

[CR1] Anme T, Segal UA (2010). Child development and childcare in Japan. Journal of Early Childhood Research.

[CR2] Centre for Community Child Health and Telethon Institute for Child Health Research (2009). A snapshot of early childhood development in Australia – AEDI National Report.

[CR3] Chen H, Keith TZ, Weiss L, Zhu J, Li Y (2010). Testing for multigroup invariance of second-order WISC-IV structure across China, Hong Kong, Macau, and Taiwan. Personality and Individual Differences.

[CR4] Cost, Quality and Child Outcomes Study Team (1995). Cost, quality and child outcomes in child care centers: Executive summary.

[CR5] Curriculum Development Council, HKSAR (2006). Guide to the pre-primary curriculum.

[CR6] Department of Health, HKSAR. & Language Information Sciences Research Centre, City University of Hong Kong (2006). Hong Kong Cantonese Oral Language Assessment Scale (HKCOLAS).

[CR7] Fernald LCH, Kariger P, Engle P, Raikes A (2009). Examining early child development in low-income countries: A toolkit for the assessment of children in the first five years of life.

[CR8] Heckman, J. J. (2004). Invest in the very young. In R. E. Tremblay, R. G. Barr, & R. DeV Peters (Eds.), *Encyclopedia on Early Childhood Development* (online). Montreal, Quebec: Centre for Excellence for Early Child Development. http://www.child-encyclopedia.com/pages/PDF/HeckmanANGxp.pdf. Accessed 1 March 2012.

[CR9] Hong Kong SAR Government (2011). Press release and publications: kindergarten education. http://www.edb.gov.hk/index.aspx?nodeID=1037&langno=1. Accessed 1 March 2012.

[CR10] Ip, P. (2012). *The Chinese early development instrument*. Unpublished manuscript, Paediatrics and Adolescent Medicine. Hong Kong: The University of Hong Kong,

[CR11] Janus M, Offord D (2007). Development and psychometric properties of the Early Development Instrument (EDI): a measure of children’s school readiness. Canadian Journal of Behavioral Science.

[CR12] Kagan SL, Britto PR (2005). Going global with indicators of child development. UNICEF Final Report.

[CR13] Kline, T. J. B. (2005). *Psychological testing: A practical approach to design and evaluation*. Thousand Oaks, CA: Sage.

[CR14] Lee K, Chiu SN, van Hasselt CA, Tong M (2009). The accuracy of parent and teacher reports in assessing the vocabulary knowledge of Chinese children with hearing impairment. Language, Speech, and Hearing Services in Schools.

[CR15] Lynch R (2004). Exceptional returns: Economic, fiscal and social benefits of investment in early childhood development.

[CR16] Magno C (2009). Demonstrating the difference between Classical Test Theory and Item Response Theory using derived test data. The International Journal of Educational and Psychological Assessment.

[CR17] Miyahara J, Meyers C (2008). Early learning and development standards in East Asia and the Pacific: Experiences from eight countries. International Journal of Early Childhood.

[CR18] Montie JE, Xiang Z, Schweinhart LJ (2006). Preschool experience in 10 countries: Cognitive and language performance at age 7. Early Childhood Research Quarterly.

[CR19] Moore AC, Akhter S, Aboud FE (2008). Evaluating an improved quality preschool program in rural Bangladesh. International Journal of Educational Development.

[CR20] Ng, S., Sun, J., & Lau, C. (in press). Early childhood education in Hong Kong: Progress, challenges and opportunities. In N. Rao, J. Zhou, & Sun, J. (Eds.), *Early childhood education in Chinese societies*. Dordrecht, Netherlands: Springer.

[CR21] NICHD Early Child Care Research Network (ECCRN) (2000). The interaction of child care and family risk in relation to child development at 24 and 36 months. Applied Developmental Science.

[CR22] NICHD Early Child Care Research Network (ECCRN) (2005). Child care and child development: Results from the NICHD study of early child care and youth development.

[CR23] Opper S (1992). Hong Kong’s young children. Their preschools and families.

[CR24] Opper S (1996). Hong Kong’s young children. Their early development and learning.

[CR25] Pang, D., & Lau, M. (2007). *Gap wider between rich and poor*. The Standard, June 19. http://www.thestandard.com.hk/news_detail.asp?pp_cat=11&art_id=47186&sid=14117720&con_type=1. Accessed 3 March 2012.

[CR26] Rao N (2006). Final Report: Vietnam early learning and development checklist.

[CR27] Rao N (2008). Final Report: Protocol to assess Lao school readiness competencies.

[CR28] Rao N (2010). Educational policy, kindergarten curriculum guidelines and the quality of teaching and learning: Lessons from kindergartens in Hong Kong. International Journal of Early Childhood Education.

[CR29] Rao N, Li H (2009). Quality matters: Early childhood education policy in Hong Kong. Early Child Development and Care.

[CR30] Rao, N., & Pearson, E. (2007). *An evaluation of early childhood care and education programmes in Cambodia.* Cambodia: UNICEF. http://www.unicef.org/evaldatabase/index_45249.html. Accessed 1 March 1 2012.

[CR31] Rao N, Sun J (2010). Early childhood care and education in the Asia-Pacific region: Moving towards Goal 1.

[CR32] Rao N, Sun J (2011). Scaling-up early childhood programs: Moving towards evidence-based decision making in the Asian region. ISSBD Bulletin. Special Section on Intersections between Research and Social Policy.

[CR33] Rouse C, Brooks-Gunn J, McLanahan S (2005). Introducing the issue: closing the achievement gap. The Future of Children.

[CR34] Royal Government of Cambodia (2007). Validation of early learning and development standards.

[CR35] Shonkoff JP, Phillips DA (2000). From neurons to neighborhoods: The science of early childhood development.

[CR36] Sirin S (2005). Socioeconomic status and academic achievement: a meta-analytic review of research. Review of Educational Research.

[CR37] Sun J, Rao N, Engle PL (2012). Assessing early child development in the East Asia Pacific region: Cultural appropriateness and item equivalence in measurement. Unpublished manuscript.

[CR38] Sylva K, Siraj-Blatchford I, Taggart B, Sammons P, Melhuish E, Elliot K, Totsika V (2006). Capturing quality in early childhood through environmental rating scales. Early Childhood Research Quarterly.

[CR39] Yeung WJ, Linver MR, Brooks-Gunn J (2002). How money matters for young children’s development: Parental investment and family processes. Child Development.

